# Mesenchymal stem cell-derived extracellular vesicles alter disease outcomes via endorsement of macrophage polarization

**DOI:** 10.1186/s13287-020-01937-8

**Published:** 2020-09-29

**Authors:** Jiangmei Wang, Jie Xia, Ruoqiong Huang, Yaoqin Hu, Jiajie Fan, Qiang Shu, Jianguo Xu

**Affiliations:** The Children’s Hospital of Zhejiang University School of Medicine and National Clinical Research Center for Child Health, 3333 Binsheng Road, Hangzhou, 310051 Zhejiang China

**Keywords:** Mesenchymal stromal cells, Extracellular vesicles, Macrophage polarization, M2 macrophages, Disease outcomes

## Abstract

Mesenchymal stem cells (MSCs) are adult stromal cells that reside in virtually all postnatal tissues. Due to their regenerative and immunomodulatory capacities, MSCs have attracted growing attention during the past two decades. MSC-derived extracellular vesicles (MSC-EVs) are able to duplicate the effects of their parental cells by transferring functional proteins and genetic materials to recipient cells without cell-to-cell contact. MSC-EVs also target macrophages, which play an essential role in innate immunity, adaptive immunity, and homeostasis. Recent studies have demonstrated that MSC-EVs reduce M1 polarization and/or promote M2 polarization in a variety of settings. In this review, we discuss the mechanisms of macrophage polarization and roles of MSC-EV-induced macrophage polarization in the outcomes of cardiovascular, pulmonary, digestive, renal, and central nervous system diseases. In conclusion, MSC-EVs may become a viable alternative to MSCs for the treatment of diseases in which inflammation and immunity play a critical role.

## Background

Mesenchymal stem (stromal) cells (MSCs) are present in a variety of tissue sources and possess multi-lineage differentiation potential. Due to their angiogenic, anti-apoptotic, regenerative, and immunomodulatory properties, a variety of clinical trials including the work from our team [1] have studied the therapeutic applications of MSCs for a wide range of diseases [2, 3]. More recently, MSCs have been tested for the treatment of coronavirus disease 2019 (COVID-19) [4]. At present, there are two registered studies examining MSC-derived extracellular vesicles (MSC-EVs) as a candidate for treating severe COVID-19 (NCT04491240, NCT04276987) [5]. The beneficial effects of MSCs were originally attributed to their ability to home to the damaged tissues and differentiate into many cell types. However, further studies demonstrated that only a small percentage of the transplanted MSCs actually engrafted in target tissues [6]. Based on the findings that conditioned medium from MSCs mimicked the effects of whole cells, it was proposed that the paracrine-secreted elements from MSCs were accountable for the mechanism [7]. These secreted elements are collectively referred to as secretome, which is composed of cytokines, growth factors, MSC-EVs, etc. [8]. Recent studies demonstrated that MSC-EVs separated from secretome were able to replicate the effects of MSCs, indicating a major role of MSC-EVs in the paracrine mechanism [9]. Potter et al. showed that both MSC-EVs and MSCs attenuated lung vascular permeability in a mouse model of hemorrhagic shock and trauma. However, MSC secretome but not MSC-EVs decreased thrombin-induced endothelial cell permeability in vitro, indicating differences in the molecular mechanisms between MSCs and MSC-EVs [10]. Carreras-Planella et al. reported that the immunomodulatory effect of MSC secretome on B cells was not mediated by MSC-EVs, but rather by protein-enriched fraction [11]. In addition, Mitchell et al. found that MSC secretome and MSC-EVs acted synergistically to stimulate muscle generation in a cardiotoxin-induced muscle injury model [12]. The present review article will describe the general properties of MSC-EVs and mechanisms of macrophage polarization and emphasize the impacts of MSC-EVs on macrophage polarization in diseases of cardiovascular, pulmonary, digestive, renal, and central nervous systems.

**Table 1:** Studies demonstrating the effects of MSC-EVs on macrophage polarization

References	Sources of MSC-EVs	Experimental model	Mediator	Major findings
Spinosa et al. [76]	Human umbilical cord-derived MSC-EVs	Mouse elastase-induced model of abdominal aortic aneurysm	miR-147	MSC-EVs with miR-147 overexpression reduced macrophage infiltration in vivo and levels of proinflammatory cytokines in coculture of aortic tissue explants and macrophages treated with elastase.
Lv et al. [77]	Rat BM-derived MSC-EVs	Rat model of myocardial infarction		MSC-EVs incorporated with hydrogel reduced apoptosis of cardiomyocytes in the border zone and enhanced M2 polarization of macrophages in the infarcted zone.
Xu et al. [78]	Rat BM-derived MSC-EVs	Mouse model of myocardial infarction		LPS-primed MSC-EVs decreased post-infarction inflammation and injury in vivo and elevated M2 macrophage polarization in vitro via AKT1/AKT2 pathway.
Sun et al. [79]	Mouse BM-derived MSC-EVs	Mouse model of dilated cardiomyopathy		MSC-EVs improved cardiac function, attenuated cardiac dilation, and elevated cardiac M2-like F4/80+CD206+macrophages via activating JAK2-STAT6 pathway.
Zhao et al. [80]	Mouse BM-derived MSC-EVs	Mouse model of heart ischemia/reperfusion injury	miR-182	MSC-EVs alleviated myocardial ischemia/reperfusion injury via transfer of miR-182, which induced M2 macrophages polarization via targeting TLR4.
Li et al. [81]	Mouse BM-derived MSC-EVs	Mouse model of atherosclerosis	let-7	MSC-EVs alleviated atherosclerosis and enhanced M2 macrophage state in the plaque via miR-let7/HMGA2/NF-κB pathway.
Morrison et al. [82]	Human BM-derived MSC-EVs	Mouse model of LPS-induced lung injury	Mitochondrial Transfer	MSCs induced M2 phenotype via EV-mediated transfer of mitochondria. Adoptive transfer of macrophages pretreated with MSC-EVs attenuated LPS-induced acute lung injury.
Li et al. [83]	Mouse BM-derived MSC-EVs	Mouse model of lung ischemia/reperfusion injury	miR-21-5p	MSC-EVs attenuated ischemia/reperfusion injury and M1 polarization of alveolar macrophages in the lung by transferring miR-21-5p.
Wang et al. [84]	Human adipose-derived MSC-EVs	Mouse model of LPS-induced lung injury	miR-27a-3p	MSC-EVs mitigated acute lung injury via transfer of miR-27a-3p to alveolar macrophages, inducing M2 macrophage polarization.
Huang et al. [85]	Human adipose-derived MSC-EVs	Mouse model of LPS-induced lung injury		Aging and young MSC-EVs have differential effects in alleviating acute lung injury and macrophage polarization.
Deng et al. [86]	Mouse BM-derived MSC-EVs	Mouse LPS-induced acute lung injury		MSC-EVs enhanced M2 macrophage polarization via inhibiting glycolysis and alleviated lung inflammation.
Monsel et al. [87]	Human BM-derived MSC-EVs	Mouse *E. coli* pneumonia	Keratinocyte growth factor	MSC-EVs promoted survival, alleviated lung inflammation, reduced bacterial load, and induced M2 phenotype via keratinocyte growth factor.
Phinney et al. [88]	Human BM-derived MSC-EVs	Mouse model of silicosis	Mitochondrial Transfer	MSC-EVs transferred mitochondria to macrophages, blocked TLR signaling in macrophages, and reduced silica-induced lung injury.
Willis et al. [26]	Human umbilical cord MSC-EVs.	Mouse bronchopulmonary dysplasia		MSC-EVs improved pulmonary development, decreased lung fibrosis, and ameliorated pulmonary vascular remodeling via modulation of lung macrophage phenotype.
Chaubey et al. [90]	Human umbilical cord MSC-EVs.	Mouse bronchopulmonary dysplasia	TSG-6	MSC-EVs attenuated injuries in lung, heart and brain. The therapeutic effects were blocked by knockdown of TSG-6 in MSC-EVs.
Lee et al. [91]	Human umbilical cord MSC-EVs	Mouse model of hypoxic pulmonary hypertension		MSC-EVs alleviated pulmonary hypertension, blocked the influx of macrophages, and reduced the expression of proinflammatory cytokines.
Klinger et al. [92]	Human BM-derived MSC-EVs	Rat model of pulmonary hypertension		MSC-EVs reversed pulmonary hypertension, which was accompanied by reduced lung macrophages and elevated ratio of M2/M1 macrophages.
Liu et al. [93]	Mouse adipose-derived MSC-EVs	Mouse LPS/GalN-induced liver injury	miR-17	MSC-EVs colocalized with hepatic macrophages, reduced NLRP3 inflammasome activation in macrophages, and ameliorated liver injury via miR-17.
Ohara et al. [94]	Human amnion-derived MSC-EVs	Rat model of nonalcoholic steatohepatitis		MSC-EVs inhibited the M1 activation of hepatic macrophages and decreased the number of hepatic macrophages and the levels of proinflammatory cytokines.
Zhang et al. [95]	Human umbilical cord-derived MSC-EVs	Mouse LPS/GalN-induced liver injury	miRNA-299-3p	MSC-EVs attenuated liver injury, activation of the NLRP3 inflammasome, and release of proinflammatory cytokines via transfer of miRNA-299-3p to macrophages.
Lu et al. [96]	Mouse BM-derived MSC-EVs	Mouse model of autoimmune hepatitis	miR-223-3p	MSC-EVs attenuated liver injury via transfer of miR-223-3p which targeted proinflammatory gene STAT3 in macrophages.
Cao et al. [97]	Mouse BM-derived MSC-EVs	Mouse model of ulcerative colitis		MSC-EVs alleviated ulcerative colitis and elevated M2 macrophages potentially via downregulating the JAK1/STAT1/ STAT6 signaling pathway.
An et al. [98]	Dog adipose-derived MSC-EVs	Mouse model of ulcerative colitis	TSG-6	MSC-EVs mitigated colitis and enhanced the macrophage polarization from M1 to M2 phenotype in the colon via TSG-6.
Eirin et al. [99]	Pig autologous adipose-derived MSC-EVs	Pig with metabolic syndrome + renal artery stenosis	IL-10	MSC-EVs attenuated renal stenosis and elevated the number of reparative M2 macrophages via IL-10.
Song et al. [100]	Pig autologous adipose-derived MSC-EVs	Pig with metabolic syndrome + renal artery stenosis		MSC-EVs from lean pigs alleviated tubular injury and fibrosis, upregulated M2 macrophages, and downregulated M1 macrophages in stenotic kidneys.
Shen et al. [101]	Mouse BM-derived MSC-EVs	Mouse ischemia/reperfusion-induced renal injury	CCR2	MSC-EVs ameliorated renal ischemia/reperfusion injury and blocked macrophage NF-*κ*B activation via CCR2.
Liu et al. [102]	Human BM-derived MSC-EVs	Mouse model of spinal cord injury	miR-216a-5p	Hypoxia-preconditioned MSC-EVs alleviated spinal cord injury and induced microglial M2 polarization via transfer miR-216a-5p which targeted TLR4 signaling cascade.
Sun et al. [103]	Human umbilical cord-derived MSC-EVs	Mouse model of spinal cord injury		MSC-EVs enhanced the locomotor functional recovery by altering the local macrophage subsets towards M2 polarization.
Li et al. [104]	Human teeth-derived MSC-EVs	Rat model of traumatic brain injury		MSC-EVs improved motor functional recovery and alleviated cortical lesion via microglia M2 polarization.
Go et al. [105]	Monkey BM-derived MSC-EVs	Rhesus monkey model of cortical injury		MSC-EVs promoted fine motor function of the hand and induced a switch of microglia from proinflammatory towards anti-inflammatory.
Lankford et al. [106]	Rat BM-derived MSC-EVs	Rat model of spinal cord injury		MSC-EVs were transferred to the site of spinal cord injury and targeted M2 macrophages at the site.
Yang et al. [107]	Human umbilical cord-derived MSC-EVs	Rat model of post-stroke cognitive impairment	CCR2	MSC-EVs with CCR2 overexpression enhanced cognitive function by promoting microglia/macrophage M2 polarization.
Shi et al. [108]	Rat BM-derived MSC-EVs	Rat model of patellar tendon injury		MSC-EVs enhanced tendon healing and blocked inflammatory responses by inducing polarization of M2 macrophages.
Shen et al. [109]	Mouse adipose MSC-EVs	Mouse model of Achilles tendon injury		IFN-γ-primed MSC-EVs alleviated tendon injury and suppressed NF-κB-induced activation of M1 macrophages.
Henao et al. [110]	Mouse adipose MSC-EVs	Mouse model of thioglycollate-induced peritonitis		MSC-EVs attenuated peritonitis and induced a M2 phenotype in peritoneal macrophages.
Song et al. [111]	Human umbilical cord-derived MSC-EVs	Mouse model of sepsis	miR-146	IL-1β primed-MSCs enhanced M2 macrophage polarization and animal survival through EV-mediated transfer of miR-146a.
Ti et al. [112]	Human umbilical cord-derived MSC-EVs	Rat model of diabetic cutaneous wound	let-7b	LPS-primed MSC-EVs enhanced wound healing and boosted M2 macrophage polarization via transfer of let-7b, which targeted TLR4/NF-κB/STAT3/AKT pathway.
Lo Sicco et al. [113]	Human adipose-derived MSC-EVs	Mouse model of cardiotoxin-induced muscle injury		MSC-EVs alleviated cardiotoxin-induced muscle injury, reduced the expression of M1 macrophage markers, and enhanced the levels of M2 macrophage markers.

*BM* bone marrow, *MSC-EVs* mesenchymal stem cells-derived extracellular vesicles, *miR* microRNA, *TLR4* toll-like receptor 4, *HMGA2* high-mobility group A2, *LPS/GalN* lipopolysaccharide and D-galactosamine, *GVHD* graft versus host disease, *NLRP3* NOD-, LRR- and pyrin domain-containing protein 3, *TSG-6* tumor necrosis factor-α-stimulated gene-6, *CCR2* C-C motif chemokine receptor-2, *TLR* Toll-like receptor, *IFN-γ* interferon gamma

## EVs

EVs are a generic term for membrane-contained particles that are naturally released by the cells and do not contain a functional nucleus according to International Society for Extracellular Vesicles (ISEV) [13]. EVs are traditionally divided into three subtypes based on the vesicle sizes and mechanisms of biogenesis: exosomes (50–150 nm diameter), microvesicles (100–1000 nm diameter), and apoptotic bodies (50–4000 nm diameter). Exosomes are formed after fusion of multivesicular bodies (MVB), a type of late endosomes in the endolysosomal pathway, with the plasma membrane. In contrast, both microvesicles and apoptotic bodies are generated by direct outward budding from the cell surface [14]. However, there is a lack of specific markers or distinctive methodologies to differentiate the three subtypes of EVs as they share overlapping size, density, and membrane proteins [15]. ISEV has now suggested to categorize EVs upon biochemical markers or size such as small EVs (< 100 nm or < 200 nm) and medium/large EVs (> 200 nm) [13].

EVs are produced by almost all types of cells and were originally thought to be a method for cells to dispose unwanted components [16]. They are now increasingly recognized as important mediators of intercellular communication and have opened up a new field of research. EVs are composed of a lipid bilayer containing proteins/peptides, lipids, and genetic material such as mRNA, microRNA (miRNA), and DNA. There are evidences to support that EVs also enclose mitochondria, ribosomes, and proteasomes [17]. These cargoes differ significantly depending on their cell of origin and are selectively sorted into EVs. It is well-established that EVs could exert the effects of parental cells via transfer of miRNA and functional proteins [18]. miRNAs are single-stranded and non-coding RNAs with 19–24 nucleotide in size. It is estimated that miRNAs may regulate up to 30% of protein-encoding genes in mammalian cells [19]. The transferred miRNAs are able to interact with target mRNAs via canonical binding, leading to target degradation or translational repression [20]. The transferred miRNAs can also use a non-canonical pathway through activation of Toll-like receptor 7 (TLR7)/Toll-like receptor-8 (TLR8) to regulate immune responses [21]. The sorting of miRNAs into EVs involves miRNA motifs such as GGCU [22] and the miRNA-associated proteins such as Argonaute 2 and Alix [23]. The sorting of proteins into EVs utilizes endosomal sorting complex required for transport (ESCRT)-dependent machinery as well as ESCRT-independent pathway [24].

## MSC-EVs

MSC-EVs display both characteristic surface markers for MSCs (CD29, CD73, and CD105) and classical markers for EVs (CD63, CD9 and CD81) [25]. MSC-EVs acquired from different sources have similar therapeutic effects, indicating comparable compositions of diverse EVs [26]. By comparison of proteomics of published MSC-EVs, a specific protein signature was identified with 22 members, involving functions such as cell adhesion [27]. However, a consensus miRNA signature among MSC-EVs from different sources has not been reported. Transfer of miRNAs and functional proteins from MSC-EVs to target cells has been reported as the mechanisms underlying the beneficial effects. For example, MSC-EVs transferred miR-223 to cardiomyocytes, resulting in downregulation of proinflammatory genes in a mouse model of polymicrobial sepsis [28]. Uptake of EVs from MSCs with miRNA-181-5p overexpression by hepatic stellate cells ameliorated liver fibrosis via activation of autophagy in mice [29]. In addition, treatment of stroke rats with MSC-EVs enriched with miR-17–92 cluster promoted functional recovery of brain via targeting PTEN [30]. As an example of functional protein transfer, MSC-EVs were taken up by endothelial cells, elevated the expression of extracellular matrix metalloproteinase inducer (EMMPRIN), and induced angiogenesis in vivo. Pro-angiogenic effect was eliminated by knockdown of EMMPRIN [31]. Additionally, MSC-EVs transferred cystinosin to the skin fibroblasts and attenuated cystine accumulation in the fibroblasts from cystinosis patients [32].

Many studies have demonstrated that MSC-EVs contributed significantly to the benefits of MSCs. MSC-EVs were as effective in suppressing autoimmunity in models of type 1 diabetes and uveoretinitis as MSCs [33]. The effect of MSC-EVs on acute kidney injury was similar to that of MSCs and was abolished by RNase treatment, indicating an RNA-dependent mechanism [34]. MSC-EVs were also as potent as MSCs in alleviating LPS-induced acute lung injury in mice. Pretreatment of MSCs with KGF siRNA partially abolished the function of MSC-EVs, suggesting the role of KGF protein expression in the underlying mechanism [35]. These observations suggest that MSC-EVs may serve as an alternative to whole*-*cell therapy. Nevertheless, some reports showed that MSC-EVs were inferior to MSCs in immunomodulatory capacity. Conforti et al. discovered that MSC-EVs had a lower ability to modulate T cell proliferation and antibody formation than MSCs [36]. In models of LPS-induced lung injury, Silva et al. reported that MSCs produced greater protection compared with MSC-EVs prepared from the same number of cells [37].

Compared with MSCs, MSC-EVs may confer several advantages. First, it is widely proposed that MSC-EVs have better safety profile than MSCs based on the reports that MSCs might have the potential to acquire chromosomal abnormalities and may even differentiate into tumor cells [38]. However, others argued that genomic stability of cultured MSCs was not a significant source of concern [39]. In addition, MSCs with or without chromosomal abnormality became senescence and did not show transformation in vitro or in vivo [40]. On the other hand, the cell-free therapy of MSC-EVs is not concern-free. A recent study documented that EVs from tumor-educated MSCs induced the differentiation of myeloid-derived suppressor cells into immunosuppressive M2 macrophages and enhanced breast cancer progression by abrogation of anti-tumor immunity [41]. Second, most MSC-EVs are able to escape lung entrapment after infusion due to their small size and accumulate at the site of injury as early as 1 h after infusion [42]. On the other hand, most of the MSCs were entrapped in the lung capillaries immediately after intravenous injection and then redistributed to other organs in a few days [43]. Third, MSC-EVs can effectively cross the plasma membrane and biological barriers such as the blood-brain barrier, providing effective delivery of immunoregulatory molecules [44]. In addition, MSC-EVs are easy to store and handle, avoiding the use of dimethyl sulfoxide for preservation.

There is an urgent need to manufacture large quantity of MSC-EVs with high quality for therapeutic application. The quality of MSC-EVs relies on the status of their parental MSCs. Overexpression of several genes or preconditioning of MSCs with cytokines, hypoxia, and stimuli has been documented to enhance the immunomodulatory and regenerative effects of MSC-EVs. Yu et al. found that EVs from GATA-4 overexpressing MSCs transferred miRNAs, such as anti-apoptotic miR-19a, to damaged cardiomyocytes and resulted in the activation of the AKT and extracellular signal-regulated kinase (ERK) signaling pathways and cell survival [45]. Domenis et al. discovered that treatment of MSCs with interferon gamma (IFN-γ) and tumor necrosis factor alpha (TNF-α) induced the release of MSC-EVs which had the capacity to switch macrophages to M2 phenotype [46]. In a mouse model of myocardial infarction, EVs from hypoxia-primed MSCs facilitated cardioprotection through prevention of cardiomyocyte apoptosis mediated by miR-125b [47]. In a mouse model of acute radiation syndrome, macrophages treated with EVs from LPS-primed MSCs secreted anti-inflammatory IL-10 and improved hematopoiesis and survival [48]. To increase manufacturing quantity, most of the existing protocols for large scale production of MSC-EVs involve multi-layer culture flasks or bioreactors aiming to maximize the culture surface areas. Recently, Haraszti et al. combined three-dimensional cultures with tangential flow filtration, a method for purifying biomolecules and EVs, and generated about 200-fold more EVs than conventional two-dimensional cultures [49].

## M1 and M2 macrophages

Macrophages are an essential component in innate and adaptive immune responses. These cells participate in inflammation, host defense, tissue-specific function, and homeostasis. Macrophages are traditionally classified into classically activated M1 phenotype and alternatively activated M2 phenotype, which is oversimplified to account for phenotypes observed under different conditions. During the past decade, many studies have revealed a spectrum model of macrophage activation states beyond the M1/M2-polarization [50, 51]. M1–M2 dichotomy was first introduced by Mills et al. to differentiate metabolism of arginine between macrophages from C57BL/6 and BALB/c mice [52]. M1 macrophages are favored by toll-like receptor (TLR) ligands (such as lipopolysaccharide, LPS) and proinflammatory cytokines such as IFN-γ and TNF-α [53]. M1 macrophages possess microbicidal activity and are characterized by enhanced ability to produce proinflammatory mediators, such as TNF-α, IL-1β, IL-6, IL-12, inducible nitric oxide synthase, and proteolytic enzymes [54]. They form the first line of defense in response to pathogens and augment Th1 polarization of CD4^+^ lymphocytes. Phenotypically, they express high levels of MHC-II, CD68, and costimulatory molecules CD80 and CD86 [55]. In contrast, M2 macrophages are at the extreme opposite of M1 spectrum and are traditionally thought to be induced by Th2 cytokines, such as IL-4 and IL-13. M2 macrophages produce anti-inflammatory cytokines, such as transforming growth factor beta **(**TGF-β), IL-1 receptor antagonist (IL-1RA), and IL-10. They participate in tissue repair, wound healing, phagocytosis, angiogenesis, and fibrosis. Phenotypically, they are characterized as IL-12^low^IL-10^high^IL-1RA^high^and show high levels of CD206, arginase 1, and CD163 [56]. To set apart from bidirectional polarization model, M2 macrophages have been further sub-divided into a spectrum of intermediate activation states: M2a, M2b, M2c, and M2d [57]. M2a is triggered by IL-4, IL-13, or infections from fungal and helminth. M2b is elicited by co-stimulation of immune complex with LPS or IL-1β, whereas M2c is induced by IL-10, TGF-β, and glucocorticoids. Finally, M2d is elicited by co-stimulation of IL-6 and adenosine [58].

## Transcriptional and post-transcriptional control of macrophage polarization

Transcription factors and post-transcriptional regulators such as miRNAs and have been reported to control macrophage polarization. Activation of transcription factors named signal transducer and activator of transcription 1 (STAT1) and nuclear factor-κB (NF-κB) p65 subunit by LPS and IFN-γ skewed macrophages towards the M1 phenotype [59]. Activation of STAT6 and NF-κB p50 homodimers by IL-4 and IL-13 favored the M2 phenotype [60]. On the other hand, activation of STAT-3 pathway through IL-10 upregulated M2 phenotype [61]. The hypoxia inducible factors (HIF) played differential roles in macrophage polarization with HIF-1α promoting the inducible nitric oxide synthase and M1 state and HIF-2α enhancing arginase 1 expression and the M2 state [62]. Additionally, Krüppel-like factor 4 (KLF4) and KLF2 promoted M2 polarization by coordinating with STAT6 and inhibiting the NF-κB/HIF-1α functions, respectively [63, 64]. Furthermore, peroxisome proliferator-activated receptor γ (PPARγ), another transcription factor, skewed M2 phenotype and regulated genes in oxidative metabolism by inhibiting NF-κB and AP-1 pathways [65].

miRNAs are able to regulate M1 and M2 macrophage polarization at the post-transcriptional level by targeting various transcription factors and adaptor proteins. In M1-polarized macrophages induced by LPS plus IFN-γ treatment, the expression of miR-181a, miR-155, miR-204, and miR-451 was upregulated, whereas the levels of miR-146a, miR-143, and miR-145 were downregulated compared with M2 polarized macrophages treated with IL-4 [66]. Thulin et al. documented that miR-9 skewed M1 polarization by targeting nuclear receptor PPARδ, resulting in inhibition of PPARδ activity and thereby preventing the B cell lymphoma-6 (BCL-6)-mediated anti-inflammatory effects [67]. Ying et al. reported that miR-127 targeted BCL-6 and downregulated its expression, which augmented the activation of c-Jun N-terminal kinase (JNK) and the development of M1 macrophages [68]. Chaudhuri et al. discovered that miR-125b overexpression potentiated M1 macrophage activation as evidenced by elevated anti-tumor immunity. Interferon regulatory factor 4 (IRF4) was a target of miR-125b in macrophages, while IRF4 knockdown mimicked the phenotype of miR-125b overexpression [69]. miR-155 expression was reduced in macrophages from AKT2 knockout mice, resulting in elevated expression of its target protein CCAAT/enhancer binding protein-β (C/EBP-β), a key regulator of M2 polarization. On the other hand, miR-155 overexpression restored the M1 phenotype in these cells [70]. Moreover, AKT1 knockout mice displayed upregulation of miR-155, M1 macrophage shift, and antibacterial response by targeting suppressor of cytokine signaling 1 (SOCS1) [71]. On the other hand, overexpression of miR-146a in wild-type macrophages suppressed IRF5, reduced inducible nitric oxide synthase, and induced M2 polarization [72]. In high-fat diet-induced adipose tissue, miR-223 promoted M2 polarization, inhibited proinflammatory activation of macrophages, and alleviated inflammatory response and insulin resistance via inhibiting Pknox1 [73]. The expression of let-7c in macrophages was reduced under LPS stimulation. Polarization of M2 phenotype was enhanced by overexpression of let-7c, which targeted C/EBP-δ, an essential transcriptional factor in inflammatory response. Conversely, knockdown of let-7c diminished M2 phenotype expression [74]. miR-27a was elevated in monocytes treated with alcohol, which was accompanied by M2 polarization. miR-27a stimulated the phosphorylation of ERK and IL-10 secretion via targeting the ERK inhibitor, sprouty2 [75].

## MSC-EV-mediated macrophage polarization in cardiovascular diseases

In a mouse elastase-induced model of abdominal aortic aneurysm, Spinosa et al. reported that miR-147, a negative regulator of macrophage inflammatory responses, was significantly elevated compared with controls. MSC-EVs with miR-147 overexpression reduced aortic diameter, inflammation, and macrophage infiltration in elastase-treated mice. MSC-EVs with miR-147 overexpression also attenuated the expression of proinflammatory cytokines in coculture of human aortic tissue explants and CD11b^+^ macrophages treated with elastase [76] (Table 1). In a rat model of myocardial infarction induced by ligation of left anterior descending coronary artery, delivery of MSC-EVs incorporated with alginate hydrogel increased their retention in the heart compared with EVs alone. Hydrogel incorporation also significantly reduced apoptosis of cardiomyocytes in the border zone and enhanced M2 polarization of macrophages in the infarcted zone. Cardiac function was significantly preserved in the hydrogel incorporation group than in EVs alone [77]. In a model of myocardial infarction of mouse, LPS-primed MSC-EVs decreased post-infarction inflammation and myocardial injury in comparison with MSC-EVs. Compared with MSC-EVs, LPS-primed MSC-EVs elevated phosphorylation activation of AKT1 and M2 polarization in vitro, while inhibiting phosphorylation of AKT2 and M1 polarization. The effect of LPS-primed MSC-EVs on macrophage polarization was partially abolished with either knocking down of AKT1 and AKT2, indicating the role of AKT1/AKT2 signaling pathway on macrophage polarization [78]. In a doxorubicin-induced dilated cardiomyopathy model, mice received bone marrow-derived MSC-EVs improved cardiac function, alleviated cardiac dilation, and attenuated cardiomyocytes apoptosis. MSC-EVs decreased cardiac M1-like F4/80^+^CD11c^+^ macrophages and elevated M2-like F4/80^+^CD206^+^ macrophages via activating JAK2-STAT6 pathway [79]. Zhao et al. documented that intramyocardial injection of MSC-EVs after ischemia/reperfusion injury decreased infarct size of the heart, an effect which was abolished by depletion of macrophages with clodronate liposomes. MSC-EVs promoted the polarization of macrophages from M1 to M2 phenotypes both in vivo and in vitro. On the other hand, M2 macrophage polarization was partially eliminated by diminishing the expression of MSC-EV miR-182, which targeted TLR4-mediated signaling pathways in macrophages [80]. In a mouse model of atherosclerosis with ApoE knockout, MSC-EVs reduced the atherosclerotic plaque area and enhanced the polarization of M2 macrophages in the plaque via miR-let7/high-mobility group A2 (HMGA2) /NF-κB pathway [81].

## MSC-EV-mediated macrophage polarization in pulmonary diseases

Morrison et al. reported that MSCs induced an M2 macrophage phenotype, which exhibited anti-inflammatory and highly phagocytic activities. MSCs modulated macrophage functions via EV-mediated transfer of mitochondria, which enhanced macrophage oxidative phosphorylation and phagocytosis. Administration of macrophages pretreated with MSC-EVs attenuated LPS-induced acute lung injury [82]. Li et al. demonstrated that miR-21-5p expression in MSC-EVs was significantly increased when MSCs were pretreated with hypoxemia/reperfusion. Intratracheal administration of MSC-EVs or miR-21-5p reduced lung ischemia/reperfusion injury, M1 polarization of alveolar macrophages, and proinflammatory cytokines [83]. Research from our group showed that MSC-EVs promoted M2 polarization in bone marrow-derived macrophages, which was blocked by anti-miR-27a-3p transduction. MSC-EVs administered systemically and intratracheally as well as MSCs had similar effects in alleviating acute lung injury, elevating alveolar macrophage miR-27a-3p, and promoting M2 macrophage polarization. miR-27a-3p targeted nuclear factor kappa B subunit 1 (NFKB1), an essential part of LPS-induced NF-κB activation. The effects of MSC-EVs on acute lung injury and M2 macrophage polarization in vivo were abolished by overexpression of anti-miR-27a-3p in MSCs [84]. Another study from our group revealed that MSC-EVs from young adults alleviated LPS-induced acute lung injury and induced M2 macrophage polarization, while aging MSC-EVs failed to exhibit the effects. In addition, the internalization of young MSC-EVs by macrophages was significantly higher compared with that of aging MSC-EVs. Furthermore, young and aging MSC-EVs had differential expression on several miRNAs relating macrophage polarization [85]. In another model of acute lung injury induced by intraperitoneal injection of LPS, MSC-EVs alleviated lung inflammation and downregulated glycolysis in the lung. In vitro, MSC-EVs promoted M2 polarization via inhibiting glycolysis in a murine alveolar macrophage cell line [86]. In a mouse model of *E.coli* pneumonia, MSC-EVs were as effective as their parental cells in improving survival, reducing lung inflammation, and decreasing bacterial load. The effects of MSC-EVs were abolished by neutralizing antibody towards keratinocyte growth factor. MSC-EVs promoted macrophage/monocyte M2 phenotypes, as evidenced by reduced inflammation and enhanced bacteria phagocytosis [87]. Phinney et al. documented that mitochondria were transferred from MSC-EVs to macrophages, enhancing the bioenergetics of acceptor macrophages. MSC-EVs blocked TLR signaling in macrophages. Administration of MSCs or MSC-EVs after silica exposure decreased the size of silicotic nodules, infiltration of inflammatory cells, and expression of proinflammatory and profibrotic genes in the lung. MSC-EVs, but not MSCs, reduced the collagen deposition in the lung [88]. In a mouse model of hypoxia-induced bronchopulmonary dysplasia, MSC-EVs improved pulmonary development, reduced lung fibrosis, and alleviated pulmonary hypertension. MSC-EVs inhibited the proinflammatory M1 state and enhanced an anti-inflammatory M2 state both in cultured macrophages and lung macrophages of the animal model [26]. In the same model of bronchopulmonary dysplasia, administration of MSC-EVs or tumor necrosis factor-α-stimulated gene-6 (TSG-6), an inducer of macrophage polarization from M1 to M2 [89], alleviated injuries in the lung, heart, and brain. Knockdown of TSG-6 in MSC-EVs abolished the therapeutic effects of EVs [90]. In a mouse model of hypoxic pulmonary hypertension, bone marrow-derived MSC-EVs alleviated vascular remodeling and pulmonary hypertension. MSC-EVs inhibited the influx of macrophages and reduced the levels of proinflammatory and pro-proliferative cytokines, such as monocyte chemoattractant protein-1 and hypoxia inducible mitogenic factor [91]. In a rat model of pulmonary hypertension induced by Sugen/hypoxia, Klinger et al. discovered that MSC-EVs reduced vascular remodeling and reverted pulmonary hypertension, which was companied by a reduction in lung macrophages and an elevation in the ratio of M2/M1 macrophages [92].

## MSC-EV-mediated macrophage polarization in digestive diseases

In a mouse model of fulminant hepatitis, MSC-EVs ameliorated LPS and D-galactosamine (LPS/GalN)-induced liver injury, colocalized with hepatic macrophages, and diminished the production of proinflammatory cytokines in macrophages. miR-17, which suppressed activation of NOD-, LRR-, and pyrin domain-containing protein 3 (NLRP3) inflammasome by targeting thioredoxin interacting protein, was abundantly expressed in MSC-EVs. Furthermore, the therapeutic effects of MSC-EVs were abolished by miR-17 inhibitor [93]. In a rat model of nonalcoholic steatohepatitis, MSC-EVs reduced the number of macrophages in the liver and the mRNA levels of proinflammatory cytokines (TNF-α, IL-1β, and IL-6). In vitro, MSC-EVs significantly blocked the M1 activation of hepatic macrophages induced by LPS [94]. Zhang et al. found that anti-inflammatory miRNA-299-3p was upregulated in EVs from MSCs pretreated with TNF-α and was transferred to macrophages. The MSC-EVs inhibited release of proinflammatory cytokines, activation of the NLRP3 inflammasome, and LPS/GalN-induced liver injury in vivo [95]. In a mouse model of autoimmune hepatitis, Lu et al. reported that MSC-EVs with miR-223-3p overexpression had more pronounced effect in attenuating liver injury. MSC-EV miR-223-3p, which targeted proinflammatory gene STAT3, was transferred to macrophages, resulting in reduced inflammatory response in vitro [96]. In a mouse model of dextran sodium sulfate-induced ulcerative colitis, MSC-EVs alleviated weight loss, disease activity index, and colon mucosa damage. MSC-EVs also increased M2 macrophages and the expression of IL-10 and TGF-β in the colon tissue potentially via downregulating the JAK1/STAT1/STAT6 signaling pathway [97]. In the same model of ulcerative colitis, An et al. found that TSG-6 in EVs mediated the protective effects on colon injury and macrophage polarization from M1 to M2 phenotype in the colon [98].

## MSC-EV-mediated macrophage polarization in renal diseases

In a porcine model of diet-induced metabolic syndrome and renal artery stenosis, Eirin et al. found that MSC-EVs administered via the stenotic renal artery colocalized with tubular cells and macrophages. MSC-EVs alleviated renal inflammation/stenosis, increased the number of reparative M2 macrophages, and upregulated expression of IL-10. Furthermore, these protective effects were abolished in pigs administered with IL-10-depleted MSC-EVs [99]. In pigs with diet-induced metabolic syndrome and renal artery stenosis, the same group also showed that MSC-EVs from pigs with metabolic syndrome were less potent than MSC-EVs from lean pigs in reducing tubular injury and fibrosis. There were significant upregulation of M2 macrophages and downregulation of M1 macrophages in stenotic kidneys treated with MSC-EVs from lean pigs [100]. Shen et al. documented that MSC-EVs had high expression of C-C motif chemokine receptor-2 (CCR2). MSC-EVs alleviated ischemia/reperfusion-induced renal injury in mice, while inhibited expression of CCR-2 abrogated the protective effect of MSC-EVs. CCR2 acted as a decoy to suppress C-C motif chemokine ligand 2 **(**CCL2)-induced NF-κB activation, a transcription factor for M1 phenotype [101].

## MSC-EV-mediated macrophage polarization in diseases of central nervous system

Liu et al. reported that hypoxia-preconditioned MSC-EVs ameliorated traumatic spinal cord injury by promoting microglia/macrophage M2 polarization. miR-216a-5p was upregulated in MSC-EVs, transferred to microglia/macrophages, and responsible for the alteration of macrophage phenotypes through TLR4/NF-κB/PI3K/AKT signaling cascades [102]. In the same model of spinal cord injury, another group documented that MSC-EVs augmented the locomotor functional recovery via downregulation of the proinflammatory cytokines, such as TNF-α, IL-6, and IFN-γ. MSC-EVs also altered the local macrophage subsets towards M2 polarization in the injured spinal cord [103]. In cultured microglia, MSC-EVs reduced neuroinflammation by shifting microglia towards M2 polarization. Administration of MSC-EVs improved motor functional recovery and alleviated cortical lesion in a rat model of traumatic brain injury [104]. In aged monkeys with cortical injury, MSC-EVs improved fine motor function of the hand. The recovery was associated with a phenotypic switch of the microglia from proinflammatory towards anti-inflammatory and homeostatic [105]. In a rat model of spinal cord injury, Lankford et al. discovered that IV-delivered MSC-EVs were detected in the injured, but not the undamaged spinal cord. Additionally, MSC-EVs selectively targeted M2 type macrophages at the site of the spinal cord injury [106]. In a rat model of post-stroke cognitive impairment achieved via transient middle cerebral occlusion, MSC-EVs with CCR2 overexpression enhanced cognitive function via oligodendrogenesis and remyelination. Microglia/macrophage M2 polarization was elevated by overexpression of CCR2, which inhibited CCL2-induced macrophage migration and activation [107].

## MSC-EV-mediated macrophage polarization in other diseases

Local administration of MSC-EVs enhanced tendon healing, inhibited inflammation and apoptotic cell accumulation, and elevated the proportion of tendon stem cells in a rat model of patellar tendon injury. There was a decrease in the proportion of CCR7+ M1 macrophages and an increase in CD163+ M2 macrophages in tendons from the MSC-EV group, which was associated with elevated expression of M2 cytokines IL-4 and IL-10 [108]. In a mouse model of Achilles tendon injury, IFN-γ-primed MSC-EVs alleviated the early inflammatory response after injury and accelerated healing. Primed MSC-EVs inhibited repair site NF-κB activity and suppressed NF-κB-induced activation of M1 macrophages [109]. MSC-EVs reduced total cells of peritoneal exudate in an experimental model of thioglycollate-elicited peritonitis, attenuated macrophage infiltration, and induced a M2 phenotype in peritoneal macrophages [110]. In a mouse model of a cecal ligation and puncture (CLP)-induced sepsis, Song et al. showed that IL-1β-primed MSCs exhibited elevated immunomodulatory properties through EV-mediated mechanisms. miR-146a was elevated by IL-1β treatment, selectively packaged into MSC-EVs, and transferred to macrophages. Both IL-1β-primed MSCs and MSC-EVs promoted macrophage M2 polarization and improved survival in septic mice. miR-146a inhibitors partially blocked the immunomodulatory properties of MSC-EVs [111]. In a rat model of diabetic cutaneous wound, Ti et al. demonstrated that LPS-primed MSC-EVs improved wound healing and modulated M2 macrophage polarization via shuttling let-7b, which targeted TLR4/NF-κB/STAT3/AKT signaling pathway [112]. In a mouse model of cardiotoxin-induced muscle injury, MSC-EVs alleviated the inflammatory response, downregulated the expression of M1 macrophage markers, elevated the levels of M2 macrophage markers, and promoted the expression of myogenic markers. EVs from MSCs exposed to hypoxic preconditioning had more dramatic effects in the muscle regeneration process [113].

## Conclusions

It has long been anticipated that MSCs might become a therapeutic tool for treating myriad diseases due to their anti-inflammatory and immunomodulatory capacities. MSC-EVs exhibit the biological effects of their parental cells via transfer of functional components to target cells. Compared with MSCs, MSC-EVs provide several advantages such as escaping lung entrapment, ability to cross biological barriers, and ease of storage. Therefore, MSC-EVs could become an attractive alternative to cell-based therapy. MSC-EVs are capable of targeting macrophages in the tissue and inducing the polarization macrophage polarization from M1 to M2 phenotype. The altered macrophage polarization accounts for many of the beneficial effects observed with administration of MSC-EVs in diseases of cardiovascular, pulmonary, digestive, renal, and central nervous systems. However, there is a gap in knowledge between MSC-EVs and skewed macrophage polarization. Foremost, it is still unclear how MSC-EVs recognize and target molecules on the surface of macrophages. Second, the routes of EV uptake by macrophages are poorly understood. Furthermore, there is a lack of consensus signature molecules for MSC-EVs due to differences in preparation methodology, culture medium, and cell sources. It remains unknown how diverse MSC-EVs result in similar changes in macrophage phenotypes. Further studies are warranted to examine the molecular mechanisms of macrophage polarization induced by MSC-EVs.

## Data Availability

Not applicable.

## Abbreviations

EVs: Extracellular vesicles
MSCs: Mesenchymal stem (stromal) cells
MSC-EVs: MSC-derived EVs
COVID-19: Coronavirus disease 2019
ISEV: International Society for Extracellular Vesicles
miRNA: MicroRNA
MVB: Multivesicular bodies
TLR: Toll-like receptor
IFN-γ: Interferon gamma
TNF-α: Tumor necrosis factor alpha
ESCRT: Endosomal sorting complex required for transport
EMMPRIN: Extracellular matrix metalloproteinase inducer
MHC: Major histocompatibility complex
TGF-β: Transforming growth factor beta
IL-1RA: IL-1 receptor antagonist
STAT1: Signal transducer and activator of transcription 1
NF-κB: Nuclear factor-κB
LPS: Lipopolysaccharide
HIF: Hypoxia inducible factor
KLF4: Krüppel-like factor 4
PPAR: Peroxisome proliferator-activated receptor
BCL-6: B cell lymphoma-6
JNK: c-Jun N-terminal *kinase*
IRF4: Interferon regulatory factor 4
C/EBP: CCAAT/enhancer binding protein
SOCS1: Suppressor of cytokine signaling 1
ERK: Extracellular signal-regulated kinase
HMGA2: High-mobility group A2
NFKB1: Nuclear factor kappa B subunit 1
LPS/GalN: Lipopolysaccharide and D-galactosamine
NLRP3: NOD-, LRR- and pyrin domain-containing protein 3
CCR2: C-C motif chemokine receptor-2
CCL2: C-C motif chemokine ligand 2
